# On Surgical Analgesia by Spinal Cocainisation
1A paper read before the Monmouthshire Division of the British Medical Association, at Abergavenny, February 26th, 1904.


**Published:** 1904-09

**Authors:** William Jones Greer


### ON SURGICAL ANALGESIA BY SPINAL COCAINISATION.[1]

BY

WILLIAM JONES GREER, F.R.C.S.I., D.P.H.I.

THIS method of producing surgical analgesia though not generally known in England is very largely practised on the Continent. The honour of discovering it rests with Corning[2] of New York, Bier[3] of Kiel first brought it under general notice, and Tuffier[4] of the Beaujon Hospital, Paris, has established the technique on a sound basis. I recently had the honour of witnessing M. Tuffier operating under the method. It is certainly a most interesting sight to watch such an operation as that for the radical cure of hernia on a patient fully conscious, and who can assist the surgeon when asked to do so; that is to say, suppose in a case of this kind that the sac is difficult to find, the patient is directed to cough, down comes the gut and the sac is easily traced. As regards pain, there certainly cannot be any, nor does the patient exhibit the least sign of uneasiness.

Now to English operators it may appear somewhat disconcerting to have a patient, so to speak, watching himself being operated upon; still, the patient cannot very well see the operation, and he is not very likely to try, yet he can surely hear the conversation of the surgeon and his assistants and nurses.

[1] A paper read before the Monmouthshire Division of the British Medical Association, at Abergavenny, February 26th, 1904.

[2] *N. York M. J.*, 1885, xlii. 483. [3] *Wien. med. Bl.*, Aug. 17th, 1899.

[4] *L'Analgésie Chirugicale par Voie Rachidienne*, 1901.

Coming to the technique, this, to those who are acquainted with a rigid aseptic ritual, will appear simple. The patient is placed in a sitting position by preference, leaning somewhat forward, the skin of the back is very carefully prepared for about twenty minutes, then an assistant on each side indicates the highest points of the iliac crests, the operator draws an imaginary line between these points, this line crosses the back between fourth and fifth lumbar spines; at[1] a point on this line 1 cm. from the middle line, a needle is thrust forward and very slightly upwards for a distance of about 5 to 7 cm. It passes through the skin, lumbar muscles, ligamentum sub-flavum between the laminæ to dura and subarachnoid space. The absolute proof that the point of the needle is in the subarachnoid space is the escape of cerebro-spinal fluid, this comes away drop by drop and has the usual characteristics in a normal case.

The syringe charged with one centigramme of cocaine hydrochlorate dissolved in twenty drops of an isotonic fluid[2] is then attached to the needle already in position, the piston is withdrawn, and a few drops of cerebro-spinal fluid are drawn into the syringe to mix with the cocaine solution, then the mixture is injected into the subarachnoid space slowly. The needle is withdrawn and the puncture is sealed with collodion and a dressing applied. In about fifteen minutes the analgesia will have taken place. In such extra-peritoneal operations as those on the lower limb, the hip, perineum, anus, rectum, vagina, uterus, testicle, prostate, bladder, ureter, kidney, the operator can act with the greatest security.[3] For intra-peritoneal operations the method is not so satisfactory, as sickness is likely to interfere with the manipulations. Tuffier holds that with his technique the duration of the analgesia is sufficient for any operation whatever it may be.

The phenomena which appear during the course of the analgesia are tinglings, prickings, numbness of the feet and

1 The fifth, Tuffier, Stiles, Kocher; between fourth and fifth, nearer fifth, Prof. Hepburn.

2 This cocaine solution in sealed "ampoules" can be obtained from M. Robert, Pharmacien, rue de Bourgogne, Paris.

3 Tuffier.

limbs. The patient speaks of a sensation of weight in the chest and stomach. There is some anxiety and also nausea. Occasionally there may be vomiting; this latter I observed in one of the patients, but it was certainly very little, and not as much as one may see under ether or chloroform anæsthesia. During the nausea the patient is pale and may have profuse sweats. The pulse is ordinarily accelerated. I examined it frequently during the operation, and I formed the opinion that this acceleration was not due to any of the steps of the operative procedure. After the analgesia has passed off headache is an almost constant symptom; generally it is very light and disappears the following day, but it may be severe and produce insomnia which may last for forty-eight hours. There is frequently a rise of temperature in the evening of the day of operation, 38-39 C. (100-103 F.). This usually drops to normal the next day.

A point of great interest to surgeons who intend to use this method of analgesia is the observation of Kocher[1] and of Cushing,[2] that under rachi-cocainisation the wound took an unfavourable course. With some hesitation I venture to suggest that this observation is open to criticism on the lines of defect in wound technique, at any rate I have not been able to confirm it. Perhaps it will be a sufficient reply to say that M. Tuffier[3] informs me that he has carried out spinal cocainisation about two thousand times. Now the service of this distinguished surgeon is a model of asepticism, and he is not likely to adhere to a method which would produce septic wounds.

Amongst others who use the method, Villar[4] of Bordeaux records 39 cases without accident. Guinard[5] of Paris gives 50 cases also without accident. Monzon[6] of Saragossa notes 152 cases with one death on the table, due, he thinks, to a very enfeebled condition consequent on a tumour of the liver producing pyloric stenosis.

[1] Kocher, *Operative Surgery*, 1903.
[2] *Ibid.* [3] Personal communication.
[4] Congrès Français de Chirurgie, 1901.
[5] *Ibid.* [6] International Medical Congress (Madrid), 1903.

With regard to how the cocaine acts, Lenandowsky,[1] who believes that the cerebro-spinal fluid is a product of the brain and partakes of the nature of lymph, holds that the cocaine passes directly into the nerve substance through its lymph channels without the intervention of the circulation. Nicolletti[2] of Naples in an experimental research declares that cocaine in contact with nervous elements does not produce any anatomical change; he holds that it first causes a vaso-constriction to which soon succeeds a vaso-dilatation. During these changes the nerve elements suffer in their nutrition, alteration of function occurs, and amongst other things anæsthesia.

Now in examining any new method of procedure in our work it is essential to take a judicial view of both sides of a question. Here I propose to bring before you the fact that disasters have occurred under rachicocainisation. Dumont[3] reports a death, and Kocher[4] records a death from tuberculous meningitis following an injection. Reclus[5] records eight deaths out of a total of 2,000, a high mortality certainly, but these figures are all from the period which immediately followed the initiation of the method. I venture to believe that more recent records will show a rapidly diminishing roll of fatalities. At any rate these calamities demand very careful attention; they most surely insist that a surgeon who undertakes the process shall render himself thoroughly acquainted with that technique which has the least or no mortality.

One thing is, I think, clear, that is that any surgeon who is competent to operate at all is competent to carry out spinal cocainisation; to such a one the details of the aseptic ceremonial are second nature, he will exercise the minutest care in preparing the patient's skin, he will ensure the sterility of his needle and syringe, he will use a sterilised solution of cocaine, and will see that his hands are as sterile as he can get them, and that these are encased in sterile gloves. Without such precautions it is folly to undertake this method; the operator must remember that if

[1] *Ann. Surg.*, 1900, xxxii. 851.
[2] Congrès internationale de Médecine, 1900.
[3] Kocher, *Op. cit.* [4] *Ibid.* [5] *N. York M. J.*, 1901, lxxiii., 1055.

he introduces the least septic material into the subarachnoid space his patient is doomed.

In conclusion, I would draw your attention to a remarkable statement of M. Ravaut.[1] He found that the injection of pure sterilised water alone into the subarachnoid space produced all the bad symptoms of the usual cocaine method, that is a rachi-cocainisation without cocaine. The inference from this is, of course, that the cocaine must be dissolved in a fluid as similar in composition to that of cerebro-spinal fluid, and that subject to this and the other conditions laid down the method is innocuous. Tuffier thinks that it is well not to use the procedure in either infants or hysterical people. Cardiac disease or arterio-sclerosis is not any contra-indication. I have not attempted to define the place of rachi-cocainisation in anæsthesis, as Time will unveil to the light of reason that Truth is more our friend than either Plato or Socrates.

### THE RELATIONSHIP OF CHOREA AND RHEUMATISM.

[1] Quoted by Guinard, *Cong. Fran. de Chir.*, 1901.

